# Exploring the correlation between gait speed and balance in limb prosthesis users: A pilot study

**DOI:** 10.33137/cpoj.v8i1.45517

**Published:** 2025-08-21

**Authors:** A Alhuzaymi, G Fiedler

**Affiliations:** 1 Department of Physical Therapy, Majmaah University, Saudi Arabia.; 2 Department of Rehabilitation Science and Technology, University of Pittsburgh, USA.

**Keywords:** Limb Loss, Artificial Limbs, Gait speed, Balance, Fall Risk, Rehabilitation, Amputation, Prosthesis, Velocity

## Abstract

**BACKGROUND:**

Increasing balance and stability, along with efficient locomotion, is a high-priority goal of physical rehabilitation after limb loss in order to facilitate effective participation in society. Research in the general population suggests that the ability to walk fast is correlated to good performance in balance tests. However, it is unclear if and how prosthesis use influences this correlation.

**OBJECTIVE:**

Our small-sample pilot study aimed to explore whether the general relationship between walking speed and balance holds true for people with limb loss whose physical capabilities are inevitably influenced by their prosthetic devices.

**METHODOLOGY:**

Participants with any level of limb loss were recruited and asked to perform the Ten-Meter Walk Test and Narrowing Beam Walking Test. Scores in both tests were analyzed using Spearman's rank correlation coefficient.

**FINDINGS:**

The initial sample of eleven participants was reduced to eight (5 males, 3 females, mean age 52 years, mean height 171 cm, mean weight 68 kg, mean BMI 23, limb loss levels ranging from partial hand to trans-femoral amputation) after removing outliers. The mean Ten-Meter Walking velocity was 1.16 m/s, and the mean Narrowing Beam Test score was 11.38. The results indicate a medium to strong correlation between fast walking speed and high balance scores (ρ = 0.681, p = 0.063) when outliers are excluded.

**CONCLUSION:**

These findings are consistent with prior research conducted in other populations. However, outliers in our data suggest that this relationship is not universal across all individuals with limb loss. Possible confounding variables include the activity level and the respectively prescribed prosthetic technology. Our finding, that gait speed and balance scores should be evaluated separately to tailor rehabilitation strategies effectively, is preliminary and needs to be confirmed in a larger study.

## INTRODUCTION

It is estimated that 185,000 individuals in the U.S. undergo amputation each year, with the majority of cases attributed to diabetes and peripheral vascular disease.^1,2^ Limb loss can also result from traumatic injuries, including motor vehicle accidents, combat injuries, and work-related accidents.^3,4^ The removal of cancerous tissues or the treatment of certain congenital deformities may result in amputation as well.^5,6^

As an irreversible impairment of the patient's physical integrity, limb loss poses numerous challenges to activities of daily living, including participation in society and gainful employment. Postural control, for example, is a critical skill for sustaining everyday activities. Balance is the capacity to realign the center of mass within its base of support to maintain stability. Horak^7^ proposed that maintaining postural balance depends on six key sub-components: biomechanical constraints, movement strategies, sensory strategies, spatial orientation, dynamic control, and cognitive processing. Losing a limb alters both the weight distribution within the body and the individual's capacity to maintain equilibrium by adjusting body movement.^7,8^

In addition, there may be limb-loss related comorbidities that increase a patient's fall risk. Lower limb amputation surgery and subsequently reduced mobility are associated with high levels of mortality^9^ and morbidity rates, including a higher risk of developing coronary artery disease^10^ and elevated risk of psychiatric disorders.^11^ Individuals with limb loss may have limited walking ability, which is known to correlate with poor balance and may restrict activity and participation.^12^ A low activity level has been shown to be associated with muscle weakness and impaired joint proprioception.^13^ Gait biomechanics are influenced by both reduced walking speeds and lower levels of physical activity.^14,15^

Post-surgical muscle weakness, after lower or upper limb amputation, may further increase fall risk,^2^ as may side effects from prescription medication. This often compounds the elevated fall risk that is already typical for old age regardless of limb loss status.^4^ Among the elderly, fear of falling has been reported in between 20% to 46% of nonfallers and 40% to 73% among those who have recently fallen.^16–20^ By comparison, more than half of individuals with lower limb loss report a minimum of one fall within a year, with roughly about 33% experiencing multiple falls even after completing a comprehensive rehabilitation program.^3,21^

The research literature demonstrates that walking performance is directly linked with balance.^22–27^ Slow gait speed is recognized as a significant contributor to fall risk, highlighting the need to prioritize the assessment of gait speed in people with limb loss to establish comprehensive data on fall risk factors.^28,29^ Most research has focused on persons with lower limb loss who have been found to have significantly reduced both gait and cognitive performance during single-task testing and even more so during dual-task testing.^30^ Relatively little is known on how upper limb loss affects the relationship between gait speed and balance. Furthermore, it is possible that differences in prosthetic hardware affect this relationship. For instance, prosthetic foot/ankle component designs strike a compromise between dynamic efficiency (i.e., gait speed) and static stability (i.e., balance) but cannot be optimized for both at the same time. This raises the question of whether gait speed is as much a generalizable predictor of balance (or vice versa) in people with limb loss as it is in the general population.

Despite growing awareness of fall risk factors and implementation of prevention strategies, there remains room for improvement in fall prevention programs and their application in the limb loss population.^21^ Assessments of the risk of falls include both subjective and objective measures. Common clinical outcome measures include the Activities-specific Balance Confidence (ABC) scale,^31^ the Berg Balance Scale (BBS),^32^ and the Narrowing Beam Walking Test (NBWT),^33^ which have all been found valid and reliable to identify potential risk of falling in individuals with conditions that compromise balance.^33–35^

The ABC has been shown to be limited in distinguishing between persons with a transtibial and transfemoral amputation.^35^ The BBS has demonstrated a limited capacity to assess the varying degrees of fall risk in persons with lower limb loss.^34^ The NBWT has been recently introduced as a standardized test designed to assess a wide spectrum of balance capabilities. Its unique feature lies in its capacity to pose a significant challenge to those with lower limb loss, thereby providing an effective evaluation of their balance characteristics.^33,36,37^ While “off-label” uses of the NBWT in other populations may be frequently employed, there is limited research to support its clinical use in individuals with other impairments than lower limb loss.

This pilot study aimed to explore the nature of the relationship between walking speed and balance in individuals with both lower and upper limb loss. Considering that either limb loss level affects balance, we hypothesized that slower gait speed would be associated with lower NBWT scores, irrespective of the level of amputation or the used prosthetic componentry.

## METHODOLOGY

A cross-sectional study was conducted to assess the relationship between walking speed and balance in individuals with limb loss. The study was approved by the Institutional Review Board (IRB) of the University of Pittsburgh. All participants provided informed written consent prior to their involvement in the study.

Individuals with limb loss were recruited from the institution's patient registry as well as from local prosthetic clinics. All participants were screened based on the inclusion criteria that required an age of at least 18 years, absence of at least one limb, and the ability to ambulate independently. The inclusion criteria were kept deliberately broad, in accordance with the study's aim of exploring correlations across the target population, irrespective of the severity of their limb loss. In this context, a more narrowly selected sample would be not only less representative of the population but likely also be too similar in performance to allow for meaningful analysis. Potential participants were excluded if they were unable to speak and understand English to follow study instructions, had bilateral lower limb loss, significant sensory or motor neuropathy affecting ambulation, severe visual impairments (e.g., blindness), or any other condition that could affect the validity of the walking assessments.

A target sample size of 10 was informed by the purpose of this pilot study to explore whether there may be an unusual correlation of gait speed and balance that warrants further exploration. While limiting generalizability of findings, subjecting only a small sample to the study protocol is ethically motivated and is commensurate with prior research studies in the field.^38^

Given the wide initial inclusion criteria, post-hoc analyses were considered in which the most and/or least physically active participants would be excluded from analysis, in an effort to reduce heterogeneity and focus on the subsample most representative of the majority of people with limb loss. Sociodemographic data, including age, height, weight, and sex, were collected via self-report. Prosthetic-related data such as amputation level, amputation etiology, functional (K-) levels, and amputation date were obtained prior to the performance tasks. Participants were asked to wear their daily-use prostheses and to complete a 10-meter walk test (10MWT)^39^ and the NBWT.

The 10MWT was performed at preferred gait speed and repeated three times, with the mean of the three trials utilized for scoring purposes. The 10MWT is a common assessment tool used to evaluate gait speed that has a test-retest reliability of 0.97 ICC.^40–43^

The NBWT challenges the participants’ balance control by reducing step width. With maximum supervision by the investigator, the participants were instructed to cross their arms and walk along the narrowing beam. The beam is divided into four sections of 1.83 m (6 feet) each for a total length of 7.32 m, where each section has a different width, starting with 18.6 cm, and narrowing down to 8.6 cm, 4.0 cm, and 2.0 cm respectively. The beam is made of 5.0 cm thick boards and is placed on the level floor of the gait lab. The test ends after the participant steps off the beam, uncrosses their arms, or achieves the full length of the beam. The distance traversed on the beam (in feet) is recorded as the test score. The mean of three trials per participant was used for analysis.^36,37^ To assure safety, participants were closely monitored by study personnel who were positioned close enough to provide support in the event of critical stumbles during the balance trials.

Normality of the distribution of measurements was evaluated using the Shapiro-Wilk test. Spearman correlation coefficients (ρ) were calculated to analyze the correlation between the NBWT scores and 10MWT walking speeds. Spearman correlation is comparably robust against outliers and heavy-tailed distributions^44^ that are often found in small samples like the one in the present study. Cutoff levels for deeming correlations weak (ρ = 0.1), moderate (ρ = 0.3), or strong (ρ = 0.5) were determined in accordance with convention.^45^

For all analyses, the significance level was set at α = 0.1. The deviation from the more commonly used significance criterion of α = 0.05 is justified by the safety-relevant nature of the variables at play.^46^ A type-2 error (which is less likely at α = 0.1) would mean missing a clinically significant correlation between gait speed and balance performance, which could lead to treatment decisions (e.g., prosthetic prescriptions) that are less safe by ignoring this relationship. Conversely, the consequences of a type-1 error (which is less likely at α = 0.05) are less worrisome (e.g., erroneously prescribing a safer prosthesis option than needed).

## RESULTS

**Figure 1:**
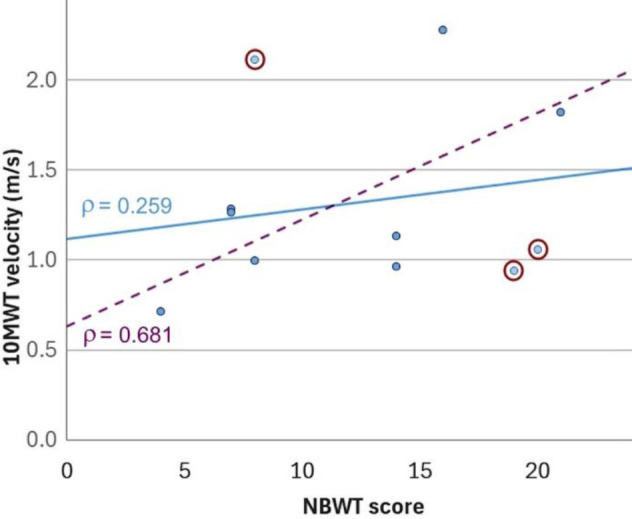
Scatter plot of 10MWT velocities (in meters per second) against NBWT scores (distance covered in feet). Correlations are shown for the full sample (continuous blue line) and the modified sample without outliers (dashed line). Outliers are circled.

A total of 11 participants fit the inclusion criteria and were included in the data collection (**Table 1**). The data (**Figure 1**) showed a mean 10MWT gait speed of 1.17 m/s (SD = 0.40 m/s) and a mean NBWT score of 12.55 (SD = 6.00) across the sample. The correlation between those variables was weak (ρ = 0.259) and not statistically significant (p = 0.442).

**Table 1:** Baseline characteristics of participants.

Variable	Mean (Standard Deviation) or Count	
	Full sample	Sample with outliers removed
Age (years)	50 (19.4)	52 (17.4)
Height (cm)	171 (8.01)	171 (8.3)
Weight (kg)	75 (4.48)	68 (8.3)
Male/female ratio	8/3 (73%/27%)	5/3 (63%/37%)
BMI^a^	25 (1.15)	23 (2.8)
Amputation type (No.)^b^	TF (1), TT (4), RP (1), TR (2), TH (2), FA (1)	TF (1), TT (4), TR (1), TH (1), FA (1)
Years since limb loss	19 (17.02)	21 (12.5)
MFCL (K-) level, (No.)^c^	K1 (0), K2 (0), K3 (9), K4 (2)	K1 (0), K2 (0), K3 (7), K4 (1)

a BMI indicates body mass index, calculated as weight in kilograms divided by height in meters squared.

b Amputation types: TF, transfemoral; TT, transtibial; RP, rotationplasty; TR, transradial; TH, transhumeral; FA, finger amputation.

c MFCL indicates Medicare Functional Classification Level; K-levels range from K1 to K4.

For a secondary analysis, data were excluded from participants who were deemed outliers unrepresentative of the general amputee population due to their activity level being uncommonly high (along with an unusual amputation level, i.e., a Van Ness Rotationplasty, n = 1) or low (signified by an age of above 70 years, n = 2). In the thus updated sample (n = 8, **Table 1**) the mean 10 MWT gait speed was 1.16 m/s (SD = 0.40 m/s) and mean NBWT scores were 11.38 (SD = 5.76). Their correlation was classified as moderate to strong (ρ = 0.681) and was significant at the 0.1 level (p = 0.063).

## DISCUSSION

This work explored the hypothesis that the correlation between gait speed and balance applies to people with limb loss. Participants performed the 10MWT and the NBWT to generate the necessary data. Our findings suggest that, by trend, participants who took longer to walk 10 meters performed worse in the NBWT, which indicates poorer balance.

This does align with previous research that has demonstrated the predictive value of gait speed on fall risk in various populations, albeit using different assessment methods.^47–49^ This is the first research study that directly correlated walking speed and NBWT scores in people with limb loss. The strength of the observed correlation was limited by the heterogeneity of our full sample, which hints at the more complex relationship between gait speed and balance in users of limb prostheses. One possible factor contributing to this complexity is that the physical performance of people with limb loss largely depends on the prostheses they use, unlike in able-bodied individuals. It is conceivable that, in the case of upper limb loss, the reduced weight of the prosthesis (if one is used at all) compared to the lost arm may pose no disadvantage when it comes to gait speed but could affect the effective response to perturbations of the user's balance. In lower limb prosthetics, gait speed and balance are influenced by prosthetic design, fit, and alignment. These factors interact differently under varying surface conditions, affecting overall stability.^50–52^

While analyzing these possible relationships was not within the scope of the pilot data collection, our data may serve as the motivation to further explore them in subsequent work.

The outliers in our sample may be explained accordingly. Two of our participants combined slow walking speed with relatively high balance scores, while one showed the opposite performance of walking fast but having poor balance. It is possible that their prosthetic design was optimized for balance at the expense of gait speed (or vice versa). While this may have been clinically indicated in these individual cases, we argue that the found compromises may not have been ideal in that they sacrificed one performance measure to benefit another. After excluding the outliers, the correlation between speed and balance increased in strength and was determined to be statistically significant.

There are other independent variables that are likely to have an effect on balance and gait speed, such as the participants’ age, cause of limb loss, prosthesis experience, and gender. These could be meaningfully investigated in a larger scale study where the sample size does not prohibit statistical corrections for multiple comparisons. An ad-hoc analysis of our sample showed a trend toward better balance relative to gait speed in individuals with upper limb loss when compared to those with lower limb loss, as indicated by a lower Speed/Balance Ratio. However, the difference was not statistically significant (p = 0.196, Cohen's d = 0.45), leaving it unresolved whether amputation level (upper or lower limb) influences balance relative to gait speed.

The clinical relevance of our findings lies in the expanded utility of the NBWT, which may be interpretable beyond its intended use as a balance assessment tool. Combining speed and balance tests may offer insights into optimization potential for prosthesis fitting and/or rehabilitation training. Somebody may have “too good” balance, if it comes at the expense of walking speed and vice versa. It may be interesting to investigate whether completion time of the NBWT is a meaningful variable in addition to the distance covered. Additionally, longitudinal studies could provide insight into whether changes in walking speed over time predict corresponding changes in NBWT performance, promising a deeper understanding of the dynamic interplay between mobility and balance.

Limitations of this study include the small sample size, especially after outliers were removed. The small sample, though typical for pilot prosthetics research,^53^ limits generalizability. A larger sample would allow to further explore the relationship between gait speed and balance in prosthesis users while also considering additional factors such as prosthetic prescription, fitness level, and neurological conditions that may influence walking speed and balance. Including upper and lower limb prosthesis users and one participant with multiple finger amputations, increased sample heterogeneity. The finger amputation may have proprioceptive balance effects, but results may differ substantially from participants with major limb loss. Future, larger-scale, studies should analyze amputation types separately, as well as different types of prosthetic hardware. There is a possible training effect that influences NBWT scores and that could not be controlled in our protocol.

## CONCLUSION

In people who use limb prostheses, it cannot be assumed that there is a universal correlation between gait speed and balance. At least in some individuals, slow walking speed is not indicative of poor balance or vice versa. To ensure valid outcome assessments in clinical care, the two variables should be monitored separately.
